# Transition to Adulthood in Pediatric Palliative Care: A Narrative Review

**DOI:** 10.3390/children11070860

**Published:** 2024-07-16

**Authors:** Franca Benini, Laura Brogelli, Anna Mercante, Luca Giacomelli

**Affiliations:** 1 Pediatric Palliative Care, Pain Service, Department of Women’s and Children’s Health, University of Padua, 35122 Padua, Italy; franca.benini@aopd.veneto.it; 2 Polistudium Srl, 20121 Milan, Italy; laura.brogelli@polistudium.it; 3 Department of Biomedical and Neuromotor Sciences, University of Bologna, 40126 Bologna, Italy; anna.mercante@unibo.it

**Keywords:** pediatric palliative care, transition, childhood, adulthood

## Abstract

Pediatric palliative care (PPC) is defined as “the active care of the child’s body, quality of life, mind and spirit, also giving support to the family”. PPC should be established once a diagnosis of life-limiting or life-threatening disease is reached and should continue as long as necessary. Therefore, pediatric palliative care (PPC) can continue for years, also given the improved care approaches for children with life-limiting or life-threatening diseases. Over time, the child may grow to become a young adult, and when this happens, the transition to adult healthcare services must be undertaken. This article discusses possible interventions, fostering an efficient transition from pediatric to adult palliative care. A narrative review presents issues, experiences, and existing programs. A “Perspectives” section presents opinions and proposals by the authors. The transition process is not limited to a change from pediatric to adult services. Rather, it includes the entire process of the development of the child and requires interdisciplinary management with proper planning and collaboration among professionals of pediatric and adult teams.

## 1. Introduction

Pediatric palliative care (PPC) is defined as the active care of the child’s body, quality of life, mind, and spirit, also giving support to the family [1,2]. PPC should be established once a diagnosis of life-limiting or life-threatening disease is reached and should continue as long as necessary. An adequate PPC plan promotes physical growth and cognitive, emotional, and social development, with a personalized program based on the analyses of the subject’s needs. It supports and improves motor, communication, sensory, cognitive, and relational functions of subjects with special health needs, where possible. The plan also includes the required supply of supports, prostheses, and environmental readjustment aids in order to facilitate the acquisition of new skills for social interaction and personal autonomy [1].

In recent years, remarkable improvements have been reached in the life expectancy of children with life-limiting and life-threatening diseases [1,3]; palliative care can continue for years while the child grows to become a young adult, and all interventions should be adapted to the patient’s changing age and needs. In these cases, the transition from PPC to adult services must be undertaken to meet multiple requirements, including care standards, linking with expert professionals, and addressing legal responsibilities [4]. The transition from pediatric to adolescent and adult services should occur within an interdisciplinary context with proper planning and collaboration among professionals and caregivers.

The transition should be a “purposeful, planned movement of adolescents and young adults with chronic physical and medical conditions from child-centered to adult-oriented healthcare systems” [5]. This period lasts from multiple months to several years. Transition is not only the referral to adult centers and the transfer of medical records, but instead, it aims at supporting the patient in gaining control over one’s healthcare, as much as the condition of the patient allows [6]. Remarkably, a structured healthcare transition (HCT) process for youth with special healthcare needs can result in improvements in adherence to care, specialized interventions, quality of life, empowerment for self-care, satisfaction with care, and healthcare utilization [7].

While the transition process has been extensively explored in several conditions, and different transition models and guidelines exist in different countries and settings [8,9,10,11,12,13,14,15,16,17,18,19,20,21,22,23,24], to our knowledge, scant literature is available on the transition process in children on PPC.

Although countries may differ in resources and specific health organizations, this article discusses possible interventions, fostering the transition to palliative care for adults, both in end-of-life as well as in chronic conditions.

This narrative review critically discusses current knowledge on the transition from PPC to adulthood palliative care, discussing evidence from other areas that may support the development of new models.

## 2. Methods

Materials for this review were searched between April and June 2024. Papers for consideration in this narrative review were identified by several interrelated PubMed searches using different combinations of pertinent keywords to be expanded (palliative care, special care, health needs, pediatric, transition, rare disease). Documents from the authors’ personal collection of literature (papers collected by the authors during many years of study and practice) were also browsed. The web was searched through Google to explore websites of organizations dealing with people with special care needs. Only papers and documents published in English were considered. Papers were selected through a discussion among all authors, based on their relevance to the topic, and without the aid of any software/AI.

## 3. Concern about Transition in PPC

Some published articles suggest that there is a growing concern about the changing needs of subjects in PPC reaching the adulthood, and specific experiences and proposed plans for an efficient transition are required. Mazzucato et al. [25] observed a threefold increase from 2006 to 2016 in the number of patients transitioning from pediatric to adult age, using data from longitudinal cohorts from the Veneto Region Rare Diseases Registry (VRRDR). These data suggest that a growing number of patients and families are facing this problem. Lotstein et al. [26], analyzing responses to a survey in the USA, found that only a few parents of children with special health needs, aged from 13 to 17 years, had discussed the child’s changing needs with their physician, had received a plan to address the issue, and had discussed seeing adult professionals.

Due to the scant or nonexistent published experiences on transition from PPC, the following sections will present information drawn from other clinical areas that may be a basis for the development of new models of transition in palliative care, from the pediatric to the adolescent and adult settings.

## 4. Barriers and Solutions

### 4.1. Barriers

This section analyzes barriers encountered by caregivers, patients, and healthcare professionals facing young people with special health needs while transitioning to adult services (Table 1).

Overall, the loss of known and familiar staff on the side of patients and caregivers, and cultural differences in healthcare and a lack of time to transfer medical records on the side of health providers, are the main barriers to an efficient transition independently of the clinical setting [6].

Gray et al. explored barriers reported by patients and health providers, finding correspondence [27]. A systematic review of barriers to correct transition in healthcare showed that relationships were the main theme in any illness group [27]. Patients are not prone to renounce the staff in pediatric institutions and build new relationships in the adult care setting. Additionally, they have problems accessing adult services and trusting unknown professionals, and they have information gaps. Sometimes, troubling beliefs and expectations toward the new professionals are present. On the side of adult professionals, these same barriers are perceived [6,28]. The authors of the systematic review [27] also conducted a focus group with interdisciplinary adult providers to better understand the perception of barriers to transition by care providers [28]. The patient’s or family’s beliefs and expectations of adult care are often different from what the provider offers, causing distrust and resistance to change. The adult professionals lamented a lack of communication with the pediatricians and limited access to medical records and histories. Finally, financial and insurance issues were reported [28].

The opinion of health providers was explored by an international survey from 20 EU countries, sent to the 77 centers of the European Reference Network for Hereditary Metabolic Disorders (MetabERN), confirming that a relevant barrier to the careful management of transition reported by pediatric metabolic physicians is lack of time. In this setting, the low availability of metabolic physicians for adults is also a serious issue. Required implementations suggested by respondents include a coordinator and a dedicated staff for transition, and specific specialty training for adult physicians [29]. Indeed, it was reported that 11% of patients are indefinitely followed up by pediatricians, only ~40% of metabolic pediatricians have received appropriate training in health issues of adolescent metabolic patients, a transition coordinator is present in ~30% of centers, and an individualized protocol for transition is present in half of centers. In almost 80% of centers, the transition is based on a medical summary, transition letter, and emergency plan shared by pediatricians with the patient and the adult professionals.

Similar themes for barriers to transition were shown by a cross-sectional survey of health providers working with youths with chronic conditions and special health needs in Philadelphia, PA: the size of the medical team, difficult access to medical records, lack of time and time-consuming tasks for the disposition of resources, cultural deficiencies in pediatric diseases, and limited resources [30].

It has been observed that common obstacles to the implementation of an efficient transition may be worsened by specific conditions, such as limited resources or race- and ethnicity-based disparities. A systematic review published in 2010 found significant differences between racial and ethnic groups and suggested that further intervention should be applied to fill gaps in transition care [31]. Although published more than ten years ago, this study is the last one in this area, and we can suspect that disparities still exist. Although race and ethnic disparities are considered by the authors as almost limited to the United States, further research is required in many countries.

### 4.2. Solutions

The literature proposes some solutions to the barriers to transition of care in children with complex conditions, including interventions aimed at improving either coordination of care or readiness to transition. This section reports examples of generalized transition models in Europe [23,32].

A randomized trial in young adults with chronic illnesses showed that implementing recommended practices of coordinated healthcare improves patients’ perceptions of receiving the needed care in a transitional period. It should be noted that only self-reported measures were used in this study [33]. Another experience of coordinated transition was reported by a group from Children’s Hospital of Philadelphia [34]. A multidisciplinary transition team consulted on 80 cases, identifying appropriate referrals. At the evaluation of this program, 78% of pediatricians caring for the referred children were satisfied with the program that helped identify adult providers, and 90% desired to use the program for future transitions. An interdisciplinary consulting service spread from this program, and it currently contributes to planning individual transition, finding adult practitioners, assisting with insurance questions, coordinating pediatric and adult hospital care, transferring the medical records, and finding general services in the community.

A 2018 review of programs for care transition found that patient readiness and pediatric/adult physicians’ cooperation are the most relevant factors for an efficient process; transition should be prepared over a long period, offering different solutions [35].

A cross-sectional study of 17,114 adolescents and young adults specifically evaluated patients’ readiness in transition [36]. Although providers encourage empowerment of one’s own health, 56% of participants had never discussed transition with their physicians, and only 35% had talked about health insurance. Based on these reports, the authors recommend preparing for transition with payor systems, depending on the local health organization. Indeed, the generalization of measures for readiness to transition is not easy, as all assessments are specific to the population of each experience, making any comparison among studies and experiences difficult [37].

Aiming at engaging adolescents in caring for their health, a Vermont health system launched a chatbot that strived to increase knowledge about conditions and medications and encourage them to deal with appointments and contacts with providers [38]. Although only a pilot study envisaged a single situation, most aims were fulfilled, showing a possible role for the tool. Patients transitioning to adulthood may benefit from personal knowledge of their own conditions and complex care needs, especially when consulting with their pediatric provider is difficult, as most experts in some clinical areas have no expertise in adolescent needs [39].

Kerr et al. conducted a systematic realist review of the literature to evaluate how intervention processes interact with contextual factors to help the transition of young adults with life-limiting diseases from pediatric to adult care [40]. Overall, reviewed studies suggest that the collaboration of pediatric and adult service providers to prepare educated personnel and necessary resources is a main factor. Additionally, early planning, encouraging autonomous decisions, and engaging with adult services are relevant. So, new plans should produce active and responsible behavior in all stakeholders, and interventions should not focus only on the organization but also on the readiness of all agents.

The same group further investigated this subject by a mixed methods realist evaluation in Ireland, envisaging a survey of health organizations and educational services for young adults in a period of transition, interviews with some young subjects, two focus groups with parents and caregivers, and interviews with providers. Two complementary types of models were identified that could facilitate transition. One focused on the staff, increasing the confidence of adult service providers to accomplish the process, and empowering professionals to train and prepare facilities. The other one focused on the young adults in developing autonomy of the subject while involving parents and caregivers. The authors suggest that models should be developed following this theory and also in light of the organization and human resources available in each context [41].

### 4.3. Exemplary Organization Models for Transition

The British National Health Service developed the Ready-Steady-Go program, which is available in several languages [42,43]. It is aimed at children and young people who have a long-term health condition and helps young people and their families prepare, plan, and move from pediatric to adult services. Young people will be transferred to adult services when they are developmentally ready, as the transition proceeds through steps adequate to one’s requirements. The program enables patients to cooperate with providers in the organization of their transition plan, and it enables young people to have confidence and to decide about their own health. This program was successfully implemented in an environment where financial barriers are not a concern, suggesting that coordination and early preparation make transition effective in this type of context.

Still, in Great Britain, a dedicated flow for the transition to adulthood has been prepared by the Together for Short Lives program [44]. The process should develop through three phases, aiming at achieving specific goals termed “ standards,” as shown in Table 2.

The Queen Nursing Institute with the Transition of Care Programme [QNI] [45] identified the nursing service goals necessary to attain each standard of the Together for Short Lives program, aiming at integrating the resource into the healthcare practice [46]. Briefly, the service goals to attain standard 1 should include offering choices that will help the young person manage her/his own care, planning for transition, information, and monitoring of the process. The service goals for standard 2 should be providing help for a change in focus, facilitating continuity of care, and supporting the identification of suitable adult services. The service goals for standard 3 include continuing the transition during times of uncertainty, providing an updated plan for the advance of care, managing pain, meeting the patient’s wishes and needs for death, and also supporting the family with care during the death. For standard 4, the service goals are ensuring that pediatric and adult services overlap up to the age of 25 years and reviewing the process with the young person. The standard 5 service goals are coordinating the services, overviewing palliative care and linking it with other specialists, involving primary care, and ensuring that professionals have the necessary skills.

The Medizinische Behandlungszentren für Erwachsene mit geistiger Behinderung oder schweren Mehrfachbehinderungen (MZEB) cares for adults with intellectual disabilities across Germany. It provides specialized clinics for adult patients with complex intellectual needs [47]. Within this frame of activity, it has a program to treat adult patients with pediatric diseases, and its wide success confirms that specified centers may efficiently coordinate pediatric and adult care in nationalized health systems.

Some countries, such as Italy [48], have no transition model for subjects with high care needs provided by a national organization. In this scenario, the main Italian scientific societies involved in pediatrics investigated the use of telemedicine in children and adolescents with chronic diseases in the transition age. They published a consensus document proposing a model facilitating the relationships between the services for the management of chronic pathologies in children and identifying the areas of application in many conditions from the first 1000 days of life to adulthood [16].

In the USA, The National Alliance to Advance Adolescent Health, based in Washington DC, provides Got Transition^®^, a national resource center on healthcare transition (HCT) [49]. It identified the six core goals of healthcare transition: create a consortium with the American Academy of Pediatrics, Catalyst Center, and Family Voices to facilitate the implementation of MCHB’s Blueprint for Change; engage patients and caregivers on the importance of a prepared transition; strengthen HCT evidence; increase adoption of evidence-based practices; enhance the role of stakeholder organizations in HCT clinical, educational, and policy improvements; and update and improve Got Transition’s website. Although the goals of Got Transition^®^ are proposed as guide strategies worldwide, many countries cannot implement these elements due to poor resources. Indeed, there is no consensus on how to address transition when resources are not available [50].

The development of models for transition plans is improving in Canada [51], France [52], and Ireland [53].

## 5. Themes Still Unaddressed in the Literature

Sexual and reproductive health in people with high care needs in the transition age is scarcely studied. Evaluation of ethical and legal challenges is important and still unanswered in this area, where people unable to give consent are concerned. Moreover, we are not aware of studies on the impact on the health of subjects after 18 years, of guardianship, living at home compared to community or long-term care settings, or the aging of families. Finally, we should answer: “What does a good life look like for your child as an adult?”.

It is possible that more experiences have addressed additional transition challenges in the USA and Europe without publication, as transition research is both time and resource-intensive, but the publication of unstandardized projects is puzzling. Additionally, solutions are often planned in view of local contingencies and are felt as difficult to generalize to an international public.

The above limitations should be taken into account when considering the findings of the present manuscript. In addition, we need to emphasize that transition approaches can hardly be investigated by means of formal studies, and therefore, all evidence is mainly based on the personal experiences of clinicians and researchers.

## 6. Perspectives

Several factors concur to increase the number of children with special health needs transitioning to adult care services, including improved survival and changing the attitudes of families. The transition process is not accomplished by identifying adult services and addressing the patient to new providers, but develops along several steps with variable goals, and implies the necessity for the healthcare system to monitor the patients’ needs and dynamically and effectively adapt its response. As a whole, the organization models for transition described above aim at the empowerment of the young person to take care of one’s own health. Thus, any program is intended to develop according to the young person’s growth and to address needs and queries when expressed. Transition is a long event, encompassing several, sometimes overlapping, phases, each one with different needs. Issues to be considered include many aspects of healthcare, such as clinical events, body growth with prosthesis changes, imbalance of body and conscience age, and new needs, such as a claim for autonomy, sexuality, changes in the approach to relationships, school integration, and continuing the dependence on a caregiver while developing autonomy.

The two models described here were elaborated in two developed and wealthy countries and are structured to address issues from a personalized perspective, relying on adequate resources. In other countries, it is important that available resources may be assessed and properly allocated to organize sustainable, lasting, and efficient transition services. Additionally, the cultural and social environment cannot be ignored, as long as social services such as schools and recreational centers will be involved in the transition of a young person to adolescent and adult services.

The authors propose that pediatric palliative care and adult health services are coordinated to address barriers in each special situation, considering each subject’s needs [1,54,55]. As a general aim, pediatric and adult services should overlap for at least 2 years. Depending on the young person’s development and available resources (in terms of not only economic availability but also of professional skills and specialization), the system should be ready either to transfer the patient to adult health services while continuing healthcare in the pediatric services for special issues (i.e., ventilation or pain care) or to maintain the patient within the pediatric care while offering support in special areas. Indeed, the physical growth is often so deficient to forbid considering these patients as adults in this single domain. Appropriate tools to evaluate the needs in the transition period, as existing for the pediatric age, should be developed to identify the most adequate setting of care to refer the young person [56]. Dedicated education should be offered to professionals of adult healthcare services to address the management of patients with rare diseases and special needs.

Social services should be involved and encouraged to develop new solutions that favor the autonomy of assisted youngsters [57]. With this aim, the school should reappraise its organization dedicated to people with disabilities to envisage personalized programs, taking into account different levels of personal capacities, relationship abilities, and physical needs. The school should provide different models of integration depending on the cognitive level and the physical impairments of the subjects. National institutions are bound to offer young people access to school, independently of their health condition, and should also favor inclusion and provide appropriate care [58]. School experiences of transitioning young people with special health needs may be frustrating and distressing if educational standards are not personalized or adequate support for physical needs is not offered [59,60,61].

Additionally, social and economic support to families and caregivers should be maintained during and after the transition period, ascertaining and meeting needs.

In conclusion, the organization of new services must be adequate to meet needs and resources [62]. The institution of clinical registers for subjects in the transition age would facilitate the assessment of needs for transition to adult services and knowing the number of subjects in the process, their needs, and characteristics. Studies evaluating clinical outcomes, organizational feasibility, economic and social impacts, efficacy, efficiency, and applicability of new strategies and models for the transition of young people with complex care needs to the adult age are necessary. Such models and strategies should respond to the patient’s and family’s needs as appropriately as possible and should ensure prolonged sustainability and equitable access. Such studies require the interest and cooperation of clinicians, policy makers, economists, educators, patients, and non-profit organizations to realistically and actively face a concern of which the scientific community has a growing awareness. Urgent answers are necessary, as the number of patients in this condition is increasing, and extemporary interventions are not adequate.

This article, through a review of the current literature, proposes a basis for the analysis of objectives, development context, and hypothesis for organization strategies, independently of different care and organization models, and available resources. This basis of analysis can promote future research and planning of transition for patients in PPC to adult services.

## Figures and Tables

**Table 1:** Barriers to an efficient transition in PPC.

Barrier	Patient/Caregiver	Heath Provider
Loss of known and familiar staff	X	
Cultural differences		X
Lack of time		X
Relationship issues	X	X
Difficult access	X	X
Troubling expectations	X	X
Distrust	X	
Lack of communication with pediatricians		X
Limited access to pediatric records		X
Lack of specialization in adult health providers		X
Size of medical team		X
Lack of resources	X	X

**Table 2:** Phases of transition according to the Together for Short Lives program [44].

Phases	Standards
1. Preparing for adulthood	1. Young people, by the age of 14, should be supported to actively approach adulthood and move to adult services. Families should be helped to change their roles.
2. Preparing to move on	2. Young people actively plan their future, are involved in ongoing multi-agency assessments, and develop a coordinated transition plan adequate to personal goals and wishes. 4. Pediatric and adult services coordinate to enable a smooth transition.
3. Settling into adult services	3. The Advance Care Plan (ACP) deals with end-of-life and ongoing care in adult services. 5. A multi-agency team supports young people and facilitates healthcare by adult services. Patients and families are informed about realistic expectations to promote confidence in the future care.

## References

[1] Benini F., Papadatou D., Bernadá M., Craig F., De Zen L., Downing J., Drake R., Friedrichsdorf S., Garros D., Giacomelli L. (2022). International standards for pediatric palliative care: From IMPaCCT to GO-PPaCS. J. Pain. Symptom Manag..

[2] WHO (2013). Strengthening of Palliative Care as a Component of Integrated Treatment throughout the Life Course. https://apps.who.int/gb/ebwha/pdf_files/EB134/B134_28-en.pdf.

[3] Making Every Young Adult Count, Estimating Current and Future Prevalence of Young People with Life-limiting and Life-Threatening Conditions in England. https://www.togetherforshortlives.org.uk/resource/making-every-young-adult-count/#:~:text=The%20Making%20Every%20Young%20Adult,with%20life%2Dlimiting%20conditions%20in.

[4] Noyes J., Pritchard S., Pritchard A., Bennett V., Rees S. (2018). Conflicting realities experienced by children with life-limiting and life-threatening conditions when transitioning to adult health services. J. Adv. Nurs..

[5] Becker J., Ravens E., Pape L., Gundula E. (2020). Somatic outcomes of young people with chronic diseases participating in transition programs: A systematic review. T Trans. Med..

[6] Berens J.C., Jan S., Szalda D., Hanna C.M. (2017). Young adults with chronic illness: How can we improve transitions to adult care?. Pediatrics.

[7] Schmidt A., Ilango S.M., McManus M.A., Rogers K.K., White P.H. (2020). Outcomes of pediatric to adult health care transition interventions: An updated systematic review. J. Pediatr. Nurs..

[8] Joshi D., Nayagam J., Clay L., Yerlett J., Claridge L., Day J., Ferguson J., Mckie P., Vara R., Pargeter H. (2024). UK guideline on the transition and management of childhood liver diseases in adulthood. Aliment. Pharmacol. Ther..

[9] Aversa T., De Sanctis L., Faienza M.F., Gambineri A., Balducci A., D’Aprile R., Di Somma C., Giavoli C., Grossi A., Meriggiola M.C. (2024). Transition from pediatric to adult care in patients with Turner syndrome in Italy: A consensus statement by the TRAMITI project. J. Endocrinol. Investig..

[10] Alter C., Boguszewski M., Clemmons D., Dobri G.A., Geffner M.E., Kelepouris N., Miller B.S., Oh R., Shea H., Yuen K.C.J. (2024). Insights from an advisory board: Facilitating transition of care into adulthood in brain cancer survivors with acquired pediatric growth hormone deficiency. Growth Horm. IGF Res..

[11] Kheir G.B., Verbakel I., Wyndaele M., Monaghan T.F., Sinha S., Larsen T.H., Van Laecke E., Birder L., Hervé F., Everaert K. (2024). Lifelong LUTS: Understanding the bladder’s role and implications across transition phases.; a comprehensive review. Neurourol. Urodyn..

[12] Davis S.A., Annis I.E., Hughes P.M., DeJong N.A., Christian R.B., Ruble L.A., Thomas K.C. (2023). Patterns of mental health service use during the transition to adulthood among autistic adolescents and young adults. Autism Adulthood.

[13] Ma J., Li J., Huang W., Wang H. (2023). Developing a theory-driven framework for a web-based intervention to improve transition in childhood cancer survivors: A protocol of realist synthesis. BMJ Open..

[14] Nguyen N.A.T., Auquier P., Beltran Anzola A., Harroche A., Castet S., Huguenin Y., Meunier S., Repesse Y., D‘Oiron R., Rauch A. (2023). Determinants of adherence and consequences of the transition from adolescence to adulthood among young people with severe haemophilia (TRANSHEMO): A multicentric French national observational cross-sectional study based on the FranceCoag registry. Haemophilia.

[15] de Beaufort C.M.C., Aminoff D., de Blaauw I., Crétolle C., Dingemann J., Durkin N., Feitz W.F.J., Fruithof J., Grano C., Burgos C.M. (2023). Transitional Care for Patients with Congenital Colorectal Diseases: An EUPSA Network Office, ERNICA, and eUROGEN Joint Venture. J. Pediatr. Surg..

[16] Esposito S., Rosafio C., Antodaro F., Argentiero A., Bassi M., Becherucci P., Bonsanto F., Cagliero A., Cannata G., Capello F. (2023). Use of Telemedicine Healthcare Systems in Children and Adolescents with Chronic Disease or in Transition Stages of Life: Consensus Document of the Italian Society of Telemedicine (SIT), of the Italian Society of Preventive and Social Pediatrics (SIPPS), of the Italian Society of Pediatric Primary Care (SICuPP), of the Italian Federation of Pediatric Doctors (FIMP) and of the Syndicate of Family Pediatrician Doctors (SIMPeF). J. Pers. Med..

[17] Mukwevho A.C., Maputle M.S., Ramathuba D.U. (2023). Growing Up with HIV: Experiences of Transition from Adolescence to Adulthood at Selected Primary Health Facilities in Limpopo Province.; South Africa. Children.

[18] Gilbert C., Bennett K.M., Brown C., Bush A. (2023). Experiences of UK-based adult transition services for interstitial lung disease in childhood: “There’s a lot less cushioning”. Pediatr. Pulmonol..

[19] Singh P., Seth A. (2023). Transition of Care of Pediatric Patients with Special Needs to Adult Care Settings: Children with Diabetes Mellitus and Other Endocrine Disorders. Indian J. Pediatr..

[20] Miyamae T., Bando Y. (2024). Transition of Patients with Childhood-onset Rheumatic Diseases to Adult Medical Care in Japan. Intern. Med..

[21] Inusa B.P.D., Stewart C.E., Mathurin-Charles S., Porter J., Hsu L.L., Atoyebi W., De Montalembert M., Diaku-Akinwumi I., Akinola N.O., Andemariam B. (2020). Paediatric to adult transition care for patients with sickle cell disease: A global perspective. Lancet Haematol..

[22] Society for Adolescent Health and Medicine (2020). Transition to Adulthood for Youth With Chronic Conditions and Special Health Care Needs. J. Adolesc. Health.

[23] Sandquist M., Davenport T., Monaco J., Lyon M.E. (2022). The Transition to Adulthood for Youth Living with Rare Diseases. Children.

[24] Bratt E.L., Mora M.A., Sparud-Lundin C., Saarijärvi M., Burström Å., Skogby S., Fernlund E., Fadl S., Rydberg A., Hanseus K. (2023). Effectiveness of the STEPSTONES Transition Program for Adolescents With Congenital Heart Disease—A Randomized Controlled Trial. J. Adolesc. Health.

[25] Mazzucato M., Visonà Dalla Pozza L., Minichiello C., Manea S., Barbieri S., Toto E., Vianello A., Facchin P. (2018). The Epidemiology of Transition into Adulthood of Rare Diseases Patients: Results from a Population-Based Registry. Int. J. Environ. Res. Public Health.

[26] Lotstein D.S., McPherson M., Strickland B., Newacheck P.W. (2005). Transition planning for youth with special health care needs: Results from the National Survey of Children with Special Health Care Needs. Pediatrics.

[27] Gray W.N., Schaefer M.R., Resmini-Rawlinson A., Wagoner S.T. (2018). Barriers to Transition from Pediatric to Adult Care: A Systematic Review. J. Pediatr. Psychol..

[28] Gray W., Dorriz P., Kim H., Partain L., Benekos E., Carpinelli A., Zupanc M., Grant K., Weiss M. (2021). Adult provider perspectives on transition and transfer to adult care: A multi-specialty.; multi-institutional exploration. J. Pediatri Nurs..

[29] Stepien K.M., Kieć-Wilk B., Lampe C., Tangeraas T., Cefalo G., Belmatoug N., Francisco R., Del Toro M., Wagner L., Lauridsen A.G. (2021). Challenges in Transition From Childhood to Adulthood Care in Rare Metabolic Diseases: Results From the First Multi-Center European Survey. Front. Med..

[30] Szalda D.E., Jimenez M.E., Long J.E., Ni A., Shea J.A., Jan S. (2015). Healthcare System Supports for Young Adult Patients with Pediatric Onset Chronic Conditions: A Qualitative Study. J. Pediatr. Nurs..

[31] Lotstein D.S., Kuo A.A., Strickland B., Tait F. (2010). The Transition to Adult Health Care for Youth with Special Health Care Needs: Do Racial and Ethnic Disparities Exist?. Pediatrics.

[32] Cook K., Siden H., Jack S., Thabane L., Browne G. (2013). Up against the System: A Case Study of Young Adult Perspectives Transitioning from Pediatric Palliative Care. Nurs. Res. Pract..

[33] Lemke M., Kappel R., McCarter R., D’Angelo L., Tuchman L.K. (2018). Perceptions of Health Care Transition Care Coordination in Patients with Chronic Illness. Pediatrics.

[34] Szalda D., Steinway C., Greenberg A., Quinn S., Stollon N., Wu K., Trachtenberg S., Jan S. (2019). Developing a Hospital-Wide Transition Program for Young Adults with Medical Complexity. J. Adolesc. Health.

[35] Green Corkins K., Miller M.A., Whitworth J.R., McGinnis C. (2018). Graduation Day: Healthcare Transition from Pediatric to Adult. Nutr. Clin. Pract..

[36] McManus M.A., Pollack L.R., Cooley W.C., McAllister J.W., Lotstein D., Strickland B., Mann M.Y. (2013). Current Status of Transition Preparation among Youth with Special Needs in the United States. Pediatrics.

[37] Schwartz L.A., Daniel L.C., Brumley L.D., Barakat L.P., Wesley K.M., Tuchman L.K. (2014). Measures of Readiness to Transition to Adult Health Care for Youth with Chronic Physical Health Conditions: A Systematic Review and Recommendations for Measurement Testing and Development. J. Pediatr. Psychol..

[38] Alderman E.M., Breuner C.C., Grubb L.K., Powers M.E., Upadhya K., Wallace S.B., Committee on Adolescence (2019). Unique Needs of the Adolescent. Pediatrics.

[39] Beaudry J., Consigli A., Clark C., Robinson K.J. (2019). Getting Ready for Adult Healthcare: Designing a Chatbot to Coach Adolescents with Special Health Needs through the Transitions of Care. J. Pediatr. Nurs..

[40] Kerr H., Price J., Nicholl H., O’Halloran P. (2017). Transition from children’s to adult services for young adults with life-limiting conditions: A realist review of the literature. Int. J. Nurs. Stud..

[41] Kerr H., Price J., Nicholl H., O’Halloran P. (2018). Facilitating transition from children’s to adult services for young adults with life-limiting conditions (TASYL): Programme theory developed from a mixed methods realist evaluation. Int. J. Nurs. Stud..

[42] https://www.readysteadygo.net/home.html.

[43] Nagra A., McGinnity P.M., Davis N., Salmon A.P. (2015). Implementing Transition: Ready Steady Go. Arch. Dis. Child. Educ. Pract. Ed..

[44] Together for Short Lives. www.togetherforshortlives.org.uk/resource/transition-adult-services-pathway.

[45] Queen Nursing Institute with the Transition of Care Programme. https://qni.org.uk/nursing-in-the-community/from-child-to-adult.

[46] McCallum C. (2017). Supporting young people’s transition from children’s to adult services in the community. Br. J. Community Nurs..

[47] Sappok T., Diefenbacher A., Winterholler M. (2019). The Medical Care of People with Intellectual Disability. Dtsch. Ärztebl. Int..

[48] Van Lierde A., Menni F., Bedeschi M.F., Natacci F., Guez S., Vizziello P., Costantino M.A., Lalatta F., Esposito S. (2013). Healthcare transition in patients with rare genetic disorders with and without developmental disability: Neurofibromatosis 1 and Williams–Beuren syndrome. Am. J. Med. Genet. Part. A.

[49] The National Alliance to Advance Adolescent Health Six Core Elements of Health Care Transition. www.gottransition.org/six-core-elements/.

[50] Narla N.P., Ratner L., Bastos F.V., Owusu S.A., Osei-Bonsu A., Russ C.M. (2021). Paediatric to adult healthcare transition in resource-limited settings: A narrative review. BMJ Paediatr. Open.

[51] CAPHC National Transitions Community of Practice (2016). A Guideline for Transition from Paediatric to Adult Health Care for Youth with Special Health Care Needs: A National Approach. www.childhealthbc.ca/sites/default/files/caphc_transition_to_adult_health_care_guideline_may_2017.pdf.

[52] Genevaz D., Arnoux A., Marcel C., Brassier A., Pichard S., Feillet F., Labarthe F., Chabrol B., Berger M., Lapointe A.S. (2022). Transition from child to adult health care for patients with lysosomal storage diseases in France: Current status and priorities—The TENALYS study, a patient perspective survey. Orphanet J. Rare Dis..

[53] National Clinical Programme for Rare Diseases (2018). Model of Care for Transition from Paediatric to Adult Healthcare Providers in Rare Diseases. www.hse.ie/eng/about/who/cspd/ncps/rare-diseases/resources/modelof-care-for-transition-from-paediatric-to-adult-healthcare-providers-in-rare-diseases.pdf.

[54] Jarvis S.W., Roberts D., Flemming K., Richardson G., Fraser L.K. (2021). Transition of children with life-limiting conditions to adult care and healthcare use: A systematic review. Pediatr. Res..

[55] Colver A., Rapley T., Parr J.R., McConachie H., Dovey-Pearce G., Le Couteur A., McDonagh J.E., Bennett C., Hislop J., Maniatopoulos G. (2019). Facilitating the transition of young people with long-term conditions through health services from childhood to adulthood: The Transition research programme. Southampt. NIHR J. Libr..

[56] Lazzarin P., Giacomelli L., Terrenato I., Benini F., behalf of the ACCAPED Study Group (2021). A Tool for the Evaluation of Clinical Needs and Eligibility to Pediatric Palliative Care: The Validation of the ACCAPED Scale. J. Palliat. Med..

[57] Champaloux S.W., Young D.R. (2015). Childhood chronic health conditions and educational attainment: A social ecological approach. J. Adolesc. Health.

[58] Innocenti UO of R. UNICEF-IRC. Towards Inclusive Education: The Impact of Disability on School Attendance in Developing Countries. www.unicef-irc.org/publications/845-towards-inclusive-education-the-impact-of-disability-on-school-attendance-in-developing.html.

[59] Jarl J., Alriksson-Schmidt A. (2021). School outcomes of adolescents with cerebral palsy in Sweden. Dev. Med. Child. Neurol..

[60] Lum A., Wakefield C.E., Donnan B., Burns M.A., Fardell J.E., Marshall G.M. (2017). Understanding the school experiences of children and adolescents with serious chronic illness: A systematic meta-review. Child. Care Health Develop..

[61] Kaushansky D., Cox J., Dodson C., McNeeley M., Kumar S., Iverson E. (2017). Living a secret: Disclosure among adolescents and young adults with chronic illnesses. Chronic Illn..

[62] Benini F., Bellentani M., Reali L., Lazzarin P., De Zen L., Pellegatta F., Aprile P.L., Scaccabarozzi G. (2021). An estimation of the number of children requiring pediatric palliative care in Italy. Ital. J. Pediatr..

